# Utility of *Synechocystis* sp. PCC 6803 glutaredoxin A as a platform to study high-resolution mutagenesis of proteins

**DOI:** 10.3389/fpls.2013.00461

**Published:** 2013-11-15

**Authors:** David B. Knaff, Roger B. Sutton

**Affiliations:** ^1^Department of Chemistry and Biochemistry, Texas Tech UniversityLubbock, TX, USA; ^2^Center for Biotechnology and Genomics, Texas Tech UniversityLubbock, TX, USA; ^3^Department of Cell Physiology and Molecular Biophysics, Texas Tech University Health Sciences CenterLubbock, TX, USA; ^4^Center for Membrane Protein Research, Texas Tech University Health Sciences CenterLubbock, TX, USA

**Keywords:** glutaredoxin A, educational platform, mutagenesis, protein structure, tertiary, synechocystis sp. PCC 6803

## Abstract

Glutaredoxin from the cyanobacterium *Synechocystis* sp. PCC 6803 is a small protein, containing only 88 amino acids, that participates in a large number of redox reactions, serving both as an electron donor for enzyme-catalyzed reductions and as a regulator of diverse metabolic pathways. The crystal structures of glutaredoxins from several species have been solved, including the glutaredoxin A isoform from the cyanobacterium *Synechocystis* sp. PCC 6803. We have utilized the small size of *Synechocystis* glutaredoxin A and its propensity to form protein crystals that diffract to high resolution to explore a long-standing question in biochemistry; i.e., what are the effects of mutations on protein structure and function? Taking advantage of these properties, we have initiated a long-term educational project that would examine the structural and biochemical changes in glutaredoxin as a function of single-point mutational replacements. Here, we report some of the mutational effects that we have observed to date.

## Introduction

Glutaredoxin, a member of the glutaredoxin/thioredoxin superfamily, was originally discovered during the search for the electron donor for *Escherichia coli* ribonucleotide reductase (Holmgren, 1976, 1979). Later, it was determined that glutaredoxins serve not only as the electron donor for a variety of reductant-requiring enzymes, but also as essential components in other cellular processes. For example, glutaredoxins play a critical role in restoring protein function following damage by oxidative stress, as well as mediating the response to heavy metal ion induced damage (Lundstrom-Ljung and Holmgren, 1995). Plant glutaredoxins play strategic roles in signaling pathways, as they participate in the regulation of diverse metabolic pathways. They are also involved in the regulation of gene expression (Couturier et al., 2013). In the case of the glutaredoxin that is the focus of this study, glutaredoxin A from the cyanobacterium *Synechocystis* sp. PCC 6803 serves as the preferred electron donor for the 2-electron reduction of arsenate to arsenite, catalyzed by arsenate reductase (Li et al., 2003; Lopez-Maury et al., 2003).

**Figure 1:**
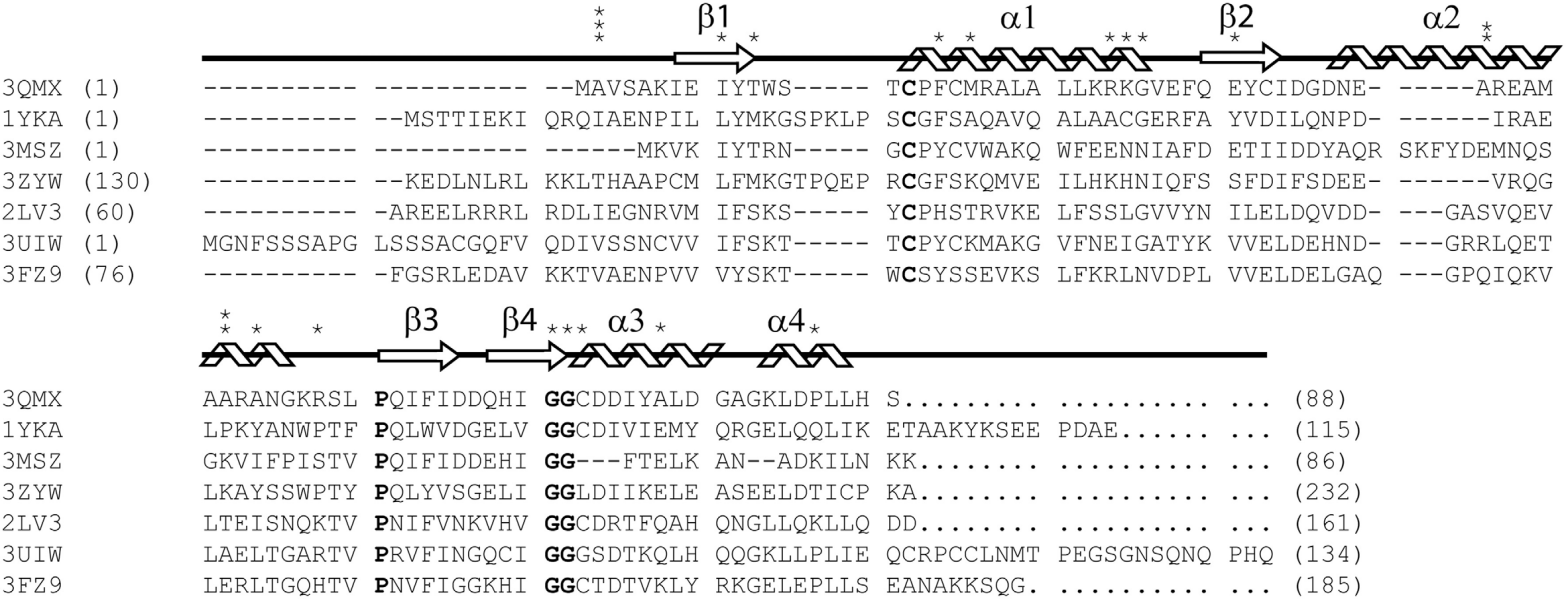
**ClustalX primary sequence alignment of *Synechocystis* sp. PCC 6803 glutaredoxin A (3QMX) Kim et al., 2012, Grx4 from *E. coli* (1YKA) (Fladvad et al., 2005), Grx1 from *Francisella tularensis* (3MSZ), glutaredoxin domain from human glutaredoxin 3 (3ZYW), Grx domain from *Mus* thioredoxin reductase (2LV3) (Dobrovolska et al., 2012), Zebrafish Grx2 (3UIW) (Brautigam et al., 2013), glutaredoxin S12 from Poplar (3FZ9) (Couturier et al., 2009).** The “*” above the sequence corresponds to mutations made and characterized within the protein. The arrow shapes represent beta-strand secondary structure; coils are regions of the protein in alpha-helical configuration. Letters in **BOLD** correspond to highly conserved features in all glutaredoxins proteins. Numbers in parentheses correspond to the residue numbers in the native protein sequence. Non-native residues were excluded from this alignment.

As part of the thioredoxin superfamily, glutaredoxin A shares the characteristic thioredoxin fold. This motif is most prominent in prokaryotic glutaredoxins, including glutaredoxin A from *Synechocystis* sp. PCC 6803, while the thioredoxin fold only exists as a substructure or domain in eukaryotic glutaredoxins (Figure 1) (Eklund et al., 1984). The small size, the ease of purification, and the physiological importance of this protein in both prokaryotes and eukaryotes make *Synechocystis* sp. PCC 6803 glutaredoxin A an ideal platform on which to base the study of protein mutations.

**Figure 2:**
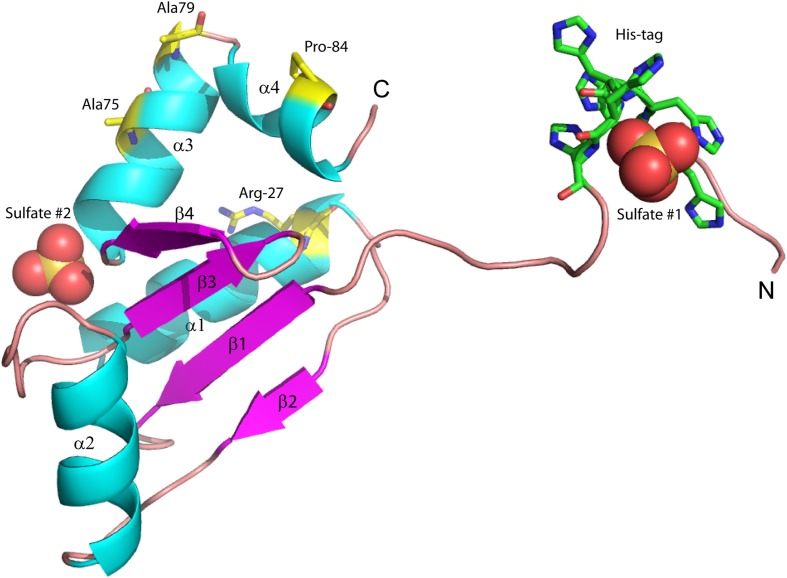
**Structure of wild-type glutaredoxin from *Synechocystis* sp. PCC 6803.** Magenta arrows correspond to β-strands; cyan coils represent α-helices. Sulfate anions are shown in the space filling representation. Also shown as sticks, are the six histidine residues used for affinity purification. The amino (N) and carboxy (C) termini are labeled.

We previously reported the high-resolution crystal structure of glutaredoxin A from *Synechocystis* sp. PCC 6803 (Figure 2) (Kim et al., 2012). The structure of the wild-type protein is comprised of the major protein secondary structure elements: four α-helical segments, and four mixed parallel: anti-parallel β-strands that form a single β-pleated sheet. The assortment of secondary structural elements and the oxidation-reduction activity of glutaredoxin are two characteristics that establish this protein as an ideal template for students to study protein structure/function in detail. The glutaredoxin that we crystallized possesses an N-terminal hexa-His affinity tag to facilitate purification of the protein. The resulting crystal structure is unusual in that the entire N-terminal His-tag extension is completely resolved and each of the histidine residues in the affinity tag is visible in the electron density. The final crystal structure includes two sulfate anions from the crystallization medium. One of the sulfate ions is coordinated at the positive end of the helix dipole of helix 3, while the other sulfate ion is coordinated to the N-terminal hexa-histidine affinity tag. The pH of the crystallization buffer is 8.0; therefore, there is likely very little charge contributed by the histidine side chains that are expected to have pK_a_ values near 6.0. The affinity of the negatively charged sulfate ion for this particular aspect of the protein probably results from a combination of the amide backbone charge interactions and shape complementarity of the N-terminus with the sulfate anion.

Another marked difference between the *Synechocystis* sp. PCC 6803 glutaredoxin A structure and other glutaredoxin structures is the lack of a disulfide bond between the two cysteine residues at the catalytically-active site, i.e., Cys15 and Cys18. The oxidation-reduction midpoint potential of the active site disulfide/dithiol couple in *Synechocystis* glutaredoxin A has been measured at −225 mV at pH 7.0 (Kim et al., 2012). Typically, glutaredoxins and thioredoxins with redox potentials in this range are found with the active site in the oxidized disulfide form at the conclusion of the purification protocol. However, the X-ray structure of *Synechocystis* glutaredoxin A unambiguously shows that the active site is fully reduced with no sign of any disulfide formation (Kim et al., 2012). The reasons for this unexpected result are not yet clear.

Site-directed mutagenesis has been used for many years to investigate the detailed function of genes and proteins (Muller et al., 1978). In this technique, the codon of a single amino acid is modified by the introduction of a mutagenic primer in a PCR amplification protocol to produce genes with alternative nucleotide sequences. When translated in a suitable expression system, these mutated genes will produce proteins with an altered primary sequence. In most mutagenesis experiments, the selection of the amino acids to be replaced is logically determined. For example, the technique is typically used to focus on specific features of a protein, such as the active sites of enzymes. In contrast, the selection of amino acids for our purposes involves choosing the mutations by random chance, without any preconceived assumption about function or location within the protein. Upon completion of this project, we will have compiled a comprehensive database of biochemical and biophysical information that includes, but is not limited to, the effects of point mutations on protein stability and activity, effects of mutation on solubility, effects of mutation on three-dimensional structure, and effects of mutation on protein crystal formation.

## Results and discussion

Widespread mutagenesis studies have previously been carried out with proteins other than glutaredoxins. For example, extensive analysis of the T4 lysozyme protein using a rational selection of sites, uncovered valuable information on the biophysics of protein folding and stability (Baase et al., 2010). However, complete coverage of mutagenesis space has not yet been accomplished for any single protein. This is largely because complete mutagenesis of a protein would require a great deal of labor, and many of the results would be scientifically trivial. In the case of glutaredoxin A, there are 88 amino acids in the complete protein, making it one of the smallest redox proteins known. If each site were mutated to one of the other 19 amino acids, 1672 new genes will have to be prepared. To accomplish this herculean task, we have integrated the research component of this project into an educational framework as an adjunct to a traditional biochemistry or protein-engineering course. A further advantage arises from the fact that the task can be accelerated by involving students from many different schools who are linked by a common database from a centralized campus via the Internet. A pilot version of this collaborative project has already collected information on protein solubility, crystallization, and structure. We have completed 26 random mutations in the three consecutive semesters that we have offered this course. Four new crystal structures have been generated.

## Effects of randomly selected mutations

Given that our mutations are randomly selected, at least four possible outcomes are expected. First, the mutated gene yields a protein that is unable to fold, and thus, would exhibit very low solubility. In this case, the protein could be sequestered in inclusion bodies in the bacteria, it could be completely proteolyzed, or it would yield only extremely low amounts of soluble protein. Second, the protein may be soluble, but is unable to crystallize under our current crystallization conditions. Third, the protein may crystallize, but the resulting crystals are too small for rapid analysis. Fourth, large, single-protein crystals are formed, and are amenable for crystallographic analysis.

We assume that many of the randomly selected mutations in the glutaredoxin gene will result in protein misfolding. For example, mutations within the hydrophobic core of most proteins are not well tolerated (Guo et al., 2004). Of the 27 mutations that we have characterized, three—T11Y, L25S, and G68Y—have resulted in the formation of an insoluble protein. The Gly68-Gly69 sequence is highly conserved within the glutaredoxin family (Figure 1). This di-glycine motif makes a bend in a loop between β-strand 4 and α-helix 3; hence, this sequence is likely to be an essential determinate of the native structure. The introduction of bulky tyrosine residues may impose considerable steric hindrance on this loop, thus, resulting in misfolding and this may explain the insolubility of the G68Y variant. In contrast, the G69F mutation is soluble, but did not crystallize. The T11Y mutation occurs at the apex of β-strand 2, adjacent to critical cysteine residues in the molecule. The introduction of a bulky side chain at this point may destabilize the core packing at this critical position.

## Mutants of glutaredoxin a that produce protein crystals

Four of our mutant proteins produced crystals suitable for X-ray structure determination (Tables 1, 2). At least 10 of the 28 mutations that we have examined have produced crystalline material (Table 1). We define “crystalline material” as protein crystals that form either thin, fibrous crystals or protein crystals that are too small to justify further effort. Proteins that form fibrous crystals typically add protein molecules to the crystal lattice preferentially along one growth axis. In our case, A2I, A2T, I9L, R27V, K28C, A43I, A43T, A49H, A49W, and R55A variants each produced sub-optimal protein crystals. Interestingly, both hydrophobic and polar variants replacing Ala2 (A2I, A2T, and A2Y) produced soluble protein, but only A2I and A2T grew protein crystals, albeit crystals of poor quality. This particular locus on the molecule is in a flexible loop at the beginning of the polypeptide chain and is not involved in any direct crystal contacts; however, there are residues from symmetry-related molecules within 5 Å of this locus. The introduction of a bulky mutation such as Tyr could add as many as 6 new amino acid neighbors within a 5 Å sphere that could interfere with crystal packing (Table 1).

**Table 1:** **List of currently studied mutations in *Synechocystis* sp. PCC 6803 glutaredoxin A**.

**Mutation**	**Soluble**	**Insoluble**	**Crystallize**	**Δ #Neighbors**	**Pseudo ΔΔG kcal/mol|prediction**		**Final structure**
WT	✓		✓	–	–		Yes
A2Y	✓		–	+6	−0.02	n	
A2I	✓		✓	+1	−0.30	n	
A2T	✓		✓	+1	−0.6	sd	
I9L	✓		✓	0	−0.59	sd	
T11Y	X	✓	–	+3	+1.39	s	
F17N	✓		–	−4	−1.89	d	
M19A	✓		–	−2	+0.91	ss	
A23N	✓		–	0	−3.62	hd	
L25S	X	✓	–	−3	−4.56	hd	
R27L	✓		✓	−2	−0.08	n	Yes
R27V	✓		✓	−1	−0.82	sd	
K28C	✓		✓	−1	+0.56	ss	
G29C	✓		–	+2	−0.44	n	
E34L	✓		–	0	+1.00	ss	
A43G	✓		–	−1	−3.54	hd	
A43I	✓		✓	+2	+0.27	n	
A43T	✓		✓	+3	−2.29	hd	
A49H	✓		✓	+2	−2.10	hd	
A49W	✓		✓	+4	−2.32	hd	
A51E	✓		–	+4	−2.84	hd	
R55A	✓		✓	−4	+0.11	n	
G68Y	X	✓	–	+1	+3.18	hs	
G69F	✓		–	+3	+1.83	s	
C70S	✓		–	0	−3.69	hd	
A75I	✓		✓	0	−1.19	d	Yes
A79S	✓		✓	0	−0.58	ds	Yes
P84R	✓		✓	+3	+1.65	s	Yes

*Soluble = mutant protein could be purified from bacterial lysate*.

*Insoluble = no mutant protein could be purified from bacterial lysate*.

Crystallize = were crystals of any quality grown?

*Δ #Neighbors = residues which could cause potential collisions in this crystallographic setting*.

*Pseudo ΔΔG (kcal/mol) n, neutral; sd, slightly destabilizing; s, stabilizing; d, destabilizing; hd, highly destabilizing; ss, slightly stabilizing; hs, highly stabilizing*.

Structure = Was a refined crystal structure produced?

**Table 2:** **Data collection and refinement statistics**.

**PDB code:**	**R27L**	**A75I**	**A79S**	**P84R**
	**4MJE**	**4MJA**	**4MJB**	**4MJC**
**DATA COLLECTION**				
Wavelength (Å)	0.9795	1.2320	1.2830	1.1270
Space group	P2_1_2_1_2_1_	P2_1_2_1_2_1_	P2_1_2_1_2_1_	P2_1_2_1_2_1_
**CELL DIMENSIONS**				
*a, b, c* (Å)	37.3, 39.1, 50.6	37.2, 38.4, 51.6	37.2, 38.1, 51.6	37.2, 38.8, 50.7
α, β, γ (°)	90, 90, 90	90, 90, 90	90, 90, 90	90, 90, 90
Resolution (Å)	30.95-1.2	30.81-2.0	30.68-2.1	30.83-1.4
Mosaicity (°)	0.35	0.67	0.4	0.79
*R*_sym_ or *R*_merge_	7.4 (30.4)	7.3 (19.2)	4.5 (13.1)	6.7 (31.9)
*I*/σ *I*	11.8 (3.3)	16 (7.0)	20.2 (2.2)	9.6 (3.2)
Completeness (%)	99.5 (99.8)	99.9 (99.7)	99.3 (87.5)	98.8 (99.8)
Redundancy	13.3 (13.2)	6.3 (6.4)	6.8 (6.6)	3.3 (3.3)
**REFINEMENT**				
Resolution (Å)	30-1.2^*^	30-2.0^**^	30.7-2.11	26.8-1.4
No. reflections	23433	5603	4418	14533
*R*_work_/*R*_free_ (%)	17.87/19.78	20.57/22.92	19.02/22.81	17.81/21.57
**NO. ATOMS**				
Protein	1654	846	838	883
SO_4_	10	–	10	5
Water	106	62	51	93
***B*-FACTORS (Å^2^)**				
protein	9.8	20.50	22.60	15.8
SO_4_	20.0	–	99.60	16.8
Water	20.0	26.40	26.90	24.1
**R.M.S. DEVIATIONS**				
Bond lengths (Å)	0.009	0.03	0.004	0.009
Bond angles (°)	1.27	2.13	0.96	1.298
**RAMACHANDRAN PLOT**				
% Ideal	97	94	95	97
% Allowed	3	6	5	3
Outliers	0	0	0	0

* *Coordinates refined with “riding” hydrogen atoms*.

** *No sulfate ions were present in this crystal structure*.

The I9L replacement should be a conservative change in the protein, as the physio-chemical properties and side chain volumes of isoleucine and leucine are very similar. Mutational studies of other proteins have concluded that these two amino acids are largely interchangeable and that replacement of one by the other generally has little or no effect on a protein's biophysical properties (Betts and Russell, 2003). Accordingly, one would predict that such a minor change should be tolerated by crystal packing. However, this mutation did affect crystal formation. The reason for this change is still unclear. K28C is another interesting mutation. In wild-type glutaredoxin, Lys28 is involved in a complex salt-bridge between α-helices 1, 2, and 3. One would expect that the substitution of an essential, conserved residue like Lys28 with a slightly polar side chain like Cys, should interfere with the biophysical properties of the molecule. However, this mutation yields soluble protein and small protein crystals.

## Four new mutant structures

Our random mutagenesis approach has yielded four new crystal structures of glutaredoxin A, one for each of the following variants: R27L, A75I, A79S, and P84R (Table 2). Each of these crystal structures provides information about the structure and function of the glutaredoxin protein, in addition to information about protein crystal formation and protein structure in general.

**Figure 3:**
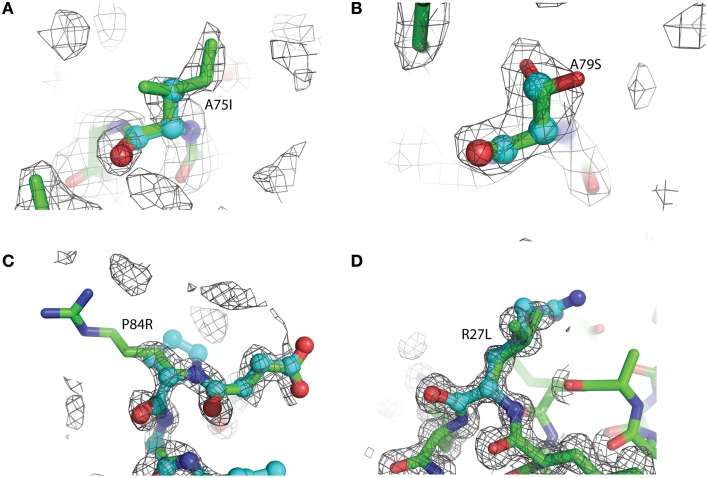
**Representative 2Fo-Fc electron density maps, contored at the 1σ level, of each of the four mutations described. (A)** A75I: The wild-type Ala is shown as light blue ball-and-stick, while the mutant Ile is shown as green sticks. The chicken wire shape around the amino acid represents the electron density carved from the final refined structure. **(B)** A79S: Ala79 is shown as blue balls-and-sticks. The mutant Ser is shown as green sticks. In this case, two conformations of Ser79 can be modeled. Both rotamers were used at 50% occupancy in the refinement of this structure. **(C)** P84R: wild-type Pro 84 is shown as blue balls-and-sticks. Mutant Arg79 is shown as green sticks. It is common for the more flexible amino acids, such as Arg or Lys, to exhibit abbreviated electron density due to the rotary motion of the side chain. **(D)** R27L: The Arg27 present in the wild-type protein is shown as blue balls-and-sticks. The structure of the leucine replacement at this position has been superimposed on top of the wild-type and is shown as green sticks.

The overall structure of the R27L variant is virtually indistinguishable from that of the wild-type protein. The RMSD (root-mean-square deviation) between both structures is 0.099 Å over all atoms, and the Leu27 substitution was confirmed in the refined experimental electron density (Figure 3). Arg27 occurs at the C-terminal end of α-helix 1 of the wild-type protein and potentially forms a H-bond with Tyr74. In addition, Arg27 occurs in a cluster of other basic residues (...K-R-K...), so it is likely that it is part of a localized positively charged patch on the surface of the protein. The leucine substitution would obviously disrupt this patch, but little else would be predicted to occur. Nevertheless, the diffraction resolution of the crystals of the R27L variant was significantly increased compared to the data obtained with wild-type glutaredoxin A. Diffraction resolution is an optical property of crystals, which is dictated by the inherent order of a crystal lattice to diffract X-rays. The lower the value for the resolution, the better the crystal is ordered and the better it can provide X-ray diffraction data that can resolve the measured distance between atoms. Crystals of the wild-type protein diffract to 1.7 Å resolution, while the R27L mutation produced crystals that diffract X-rays to 1.2 Å resolution. The sizes of the crystals of the wild-type and R27L variants were similar, so the increase in diffraction cannot be explained by larger crystals. Furthermore, as all data were collected at the same X-ray source, increased X-ray intensity cannot explain this increase in resolution. Therefore, it is likely that a property of the mutation contributed to this effect. There is a possibility that more efficient hydrophobic packing between symmetry-related molecules, mediated by the leucine replacement, contributed to more efficient crystal packing. This observation is essentially what has been described as surface entropy reduction (SER). SER is an *in silico* technique that predicts mutants, which could possibly facilitate more productive contacts among protein molecules in a crystal lattice (Goldschmidt et al., 2007). More efficient crystal packing would then result in higher X-ray scattering angles and higher overall resolution. Such a result, if it were to turn out to be a general effect, could provide information that would allow the engineering of better diffracting protein crystals. Potentially, if a low-resolution structure revealed critical crystal contacts, then mutations could be introduced to optimize those crystal contacts and increase overall diffraction without compromising the structure or activity of the protein.

Due to the location of Ala75, one might have predicted that changes in structure and function observed for the A75I and the A79S variants would be subtle. These replacements are located at the extreme C-terminus of the protein in α-helix 4 (Figure 2), and both are exposed to the outside surface of the helix. Neither is involved in crystal packing interactions nor core packing interactions, and therefore, these positions are predicted to be highly tolerant of modification. However, in the case of the A79S mutant, there are minor structural changes observed in the geometry of α-helix 4. There is a measurable decrease in the twist of the helix, a decrease in the helix-bending angle of α-helix 4 (Table 3). The significance of these minor changes remains to be explored.

**Table 3:** **Changes in helix properties in helix α-4 of glutaredoxin (Bansal et al., 2000; Kumar and Bansal, 2012)**.

**Mutation**	**Twist (°)**	***n***	**height (Å)**	**Bending angle (°)**
Wild-type	102.2	3.52	1.67	7.3
R27L	101.1	3.56	1.76	7.8
A75I	101.1	3.60	1.65	7.6
A79S	101.3	3.55	1.68	5.4
P84R	101.8	3.54	1.66	11.8

*n = amino acids/turn*.

*h (Å) = unit height of the helix*.

*Bending angle (°) = Bending angle between successive local helix axes*.

**Figure 4:**
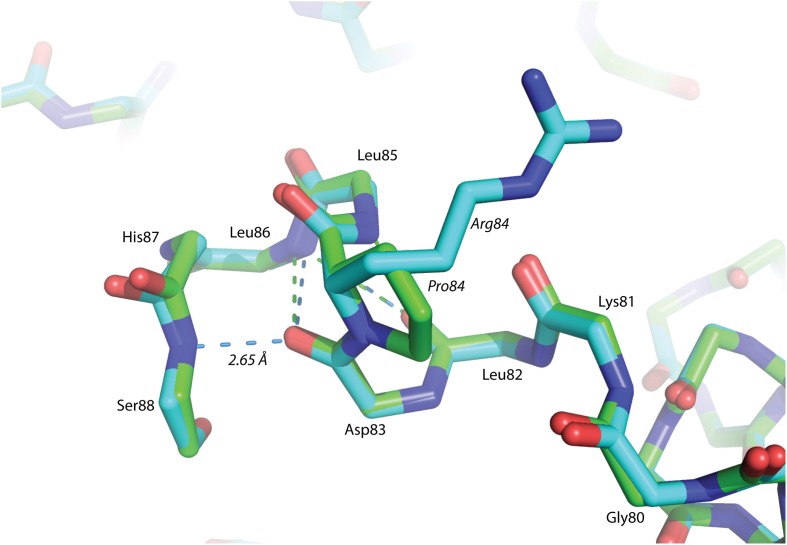
**Superposition of helix α-4 from Glutaredoxin A.** The green structure is wild-type, while the blue structure is mutant P84R. The dashed-lines are backbone H-bonds that stabilize α-helix 4. One additional consensus H-bond has been included in the P84R structure.

The P84R mutation is interesting from a structural perspective. Firstly, Pro84 occurs at the beginning of an α-helix that is bent by almost 90° relative to a neighboring α-helix in the structure. The only impetus for this dramatic bend is the maintenance of a protected hydrophobic core. A proline is present at this locus in other glutaredoxin molecules, but not all. A Blast search using the *Synechocystis* sp. PCC 6803 glutaredoxin A sequence revealed that 48% of 98 glutaredoxin sequences utilize proline at a homologous position in the primary sequence (data not shown). As proline does not provide a free backbone amide hydrogen to form an H-bond to the *i* + 4 backbone carbonyl, it rarely occurs within a helix (Kim and Kang, 1999). In the wild-type glutaredoxin structure, there is only one *i*, *i* + 4 backbone H-bond interaction to stabilize α-helix 4 (Figure 2). While mutation of Pro84 to arginine does not restore the complete backbone H-bond pattern typical of α-helices, it does appear to alleviate strain on the peptide backbone so an additional *i*, *i* + 4 backbone H-bond interaction can form between the backbone carbonyl of Asp83 and the backbone amine of Ser88(Figure 4).

## Changes in protein stability

Many of the mutations that we introduce using our random selection method could compromise protein stability in many different ways. For example, secondary structure may be disrupted by introducing a residue incompatible with β-sheet formation. The resulting misfolded protein could result in an insoluble protein in *E. coli*. To predict changes in stability, we submitted our point mutants to the Site Directed Mutator (SDM) server for analysis (Table 1). This procedure returns a prediction on whether the point mutation is neutral, stabilizing, or destabilizing based on environment-specific substitution tables and the resulting change in free energy (Worth et al., 2011). Large negative ΔΔG correlate with varying degrees of instability, while large positive ΔΔG values correlate with stabilizing energies. In general, the glutaredoxin A mutations that yielded soluble protein were predicted to be either neutral, stabilizing or destabilizing, but the range in free energies was small. The mutants at the extremes of the free energy distribution tended to yield insoluble protein. The exception to this is the C90S mutant, where the calculated free energy difference was negative (−3.69 kcal/mol), and therefore, predicted to be highly destabilizing (hd), yet the purified protein is soluble. The energetics of point mutations is clearly a complex problem; however, with more mutations, it is possible that the predictive abilities of the free energy algorithms will correlate more closely with our observations.

## Inconsistencies with the orthorhombic crystal lattice

Ideally, we would like to screen each soluble, purified glutaredoxin A mutation for its own unique crystallization condition, but we are limited in resources and by the time our students can spend with this research project. Therefore, we have concentrated on a single crystallographic condition. This restriction obviously imposes limitations on our method, but it also addresses the question about how variable or plastic a protein can be yet pack into a given lattice. Most of our mutants form suboptimal protein crystals. It is possible that the modifications we make to the glutaredoxin protein produces changes that cannot form productive crystal contacts, and therefore, disfavors efficient crystal packing. To assess this, we studied the change in the number of neighboring inter-molecular residues (within 5 Å) between mutant and wild-type glutaredoxin, assuming orthorhombic crystal packing (Table 1). More inter-molecular neighbors would likely correlate with potential clashes; whereas, fewer contacts could correlate with missing interactions. One would predict that extremes in both would make the protein less likely to crystallize in the orthorhombic setting we have measured in the wild-type glutaredoxin. Interestingly, in the case of the mutations that produced crystal structures, there is no obvious correlation between either adding or subtracting inter-molecular neighbors. The R27L crystal structure lacks two neighbors; the A75I and A79S mutant have no net differences, while the P84R mutant adds three possible interactions (Table 1). This result could reflect the plastic nature of proteins that allows them to adopt new shapes. As we have also observed that most of our soluble glutaredoxin mutants can form crystalline material, it is therefore, possible that changing the number of clashes either could make nucleation more probable or alternatively, could hinder crystal growth. As our dataset increases in size with time, we will be able to make more definitive conclusions.

## Sulfate binding

**Figure 5:**
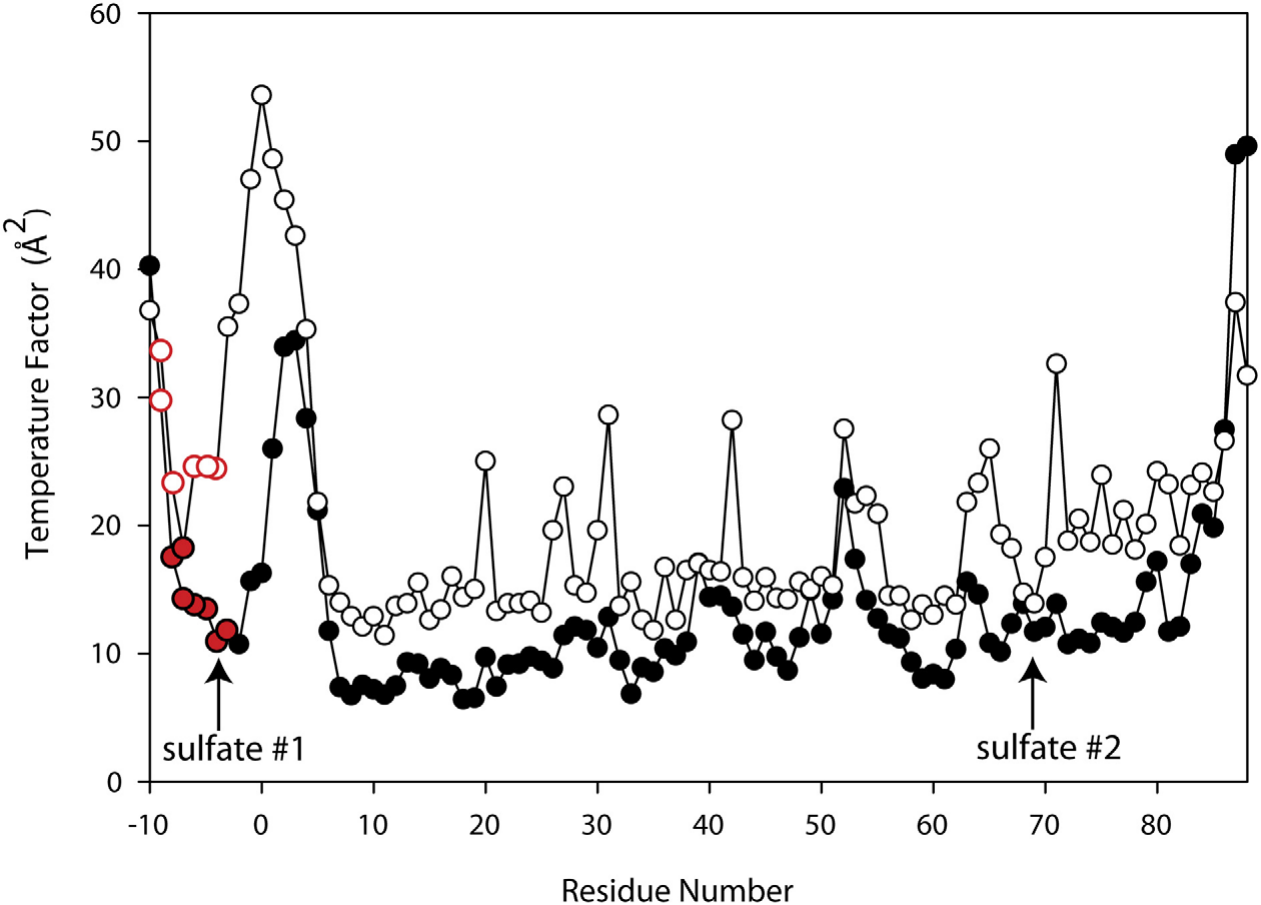
**Superimposed Temperature Factor plot of wild-type glutaredoxin (solid circles) (3QMX) vs. A75I (white circles).** The red color depicts the His-residues that make up the His-tag of the molecule. The arrows show the location of the shortest sulfate:amino acid distance.

Since ammonium sulfate is used in the crystallization medium of these glutaredoxin A crystals, there are two sulfate anions coordinated to the wild-type and to the mutated glutaredoxin A structures. Interestingly, the A75I mutation does not coordinate sulfate ions in its crystal structure. In the wild-type structure, sulfate #2 (Figure 2) is 10.6 Å from the Cα atom of Ile75, while sulfate #1 (Figure 2) is 35.2 Å distant. The large distance between the site of the mutation and the ligand binding site discounts any direct effect on sulfate binding. A cogent rationale for the absence of sulfate anions in the electron density is still unknown; however, we can draw conclusions about the effects of sulfate binding to the A75I crystal structure. As there is no sulfate anion bound to the N-terminus, the His-tag of A75I is considerably more disordered that the other glutaredoxin structures that do coordinate sulfate. The temperature factor is a measure of thermal motion within a crystal structure; atoms with higher temperature factors are thought to be more disordered. The average refined temperature-factors for the artificial N-terminus of wild-type glutaredoxin A (the loop including the His-tag residues) including the coordinated sulfate anion is 17.7 Å^2^. The same residues in the A75I structure without a coordinated sulfate anion show an average temperature factor of 32.6 Å^2^ (Figure 5). While the elevated temperature factors can be explained by the lack of sulfate coordination, the rationale for the lack of this anion in this mutation is still unknown.

## Conclusion

Our random point mutant selection scheme will ultimately cover all of the 1672 possible mutations of *Synechocystis* sp. PCC 6803 glutaredoxin A, while simultaneously teaching students about biotechnology and protein structure. There are, however, several weaknesses to this method. Due to time constraints, we have to restrict our crystallization experiments to one specific condition; one that had originally been identified during crystallization trials for the wild-type protein. While we will clearly learn how mutation affects crystal growth under these conditions, a broader-ranging, independent approach would be more informative. Another weakness of this project is that we seek to involve students from different institutions with a wide range of backgrounds and training. As such, it introduces considerable variability in the skill with which different amino acid replacements will be carried out and analyzed. While only the most diligent students will be able to make significant headway in this project, the rewards of involving a large number of students and potentially exciting them about science surely outweighs the negative aspects.

It is hoped that we will ultimately compile an extensive database that will include information to stabilize protein folds or, perhaps, to reveal techniques that improve protein crystal diffraction. However, in the limited coverage of mutational space that we have already explored, it is clear that not all mutations can be immediately understood, and their effects are still reasonably unpredictable. This is one of the primary concepts that we try to instill in students from the beginning.

## Materials and methods

### Heterologous expression

The mutagenesis protocol that we use is described in detail (Kim et al., 2012). A full description of the plasmid is described in Kim et al. (2012).

1.0 μg of plasmid DNA was added to 100 μ l of chemically competent BL-21(DE3) *E. coli* in a 1.5 ml microfuge tube. This mixture was incubated on ice for 30 min. The cells were then heat-shocked at 37°C in a water-bath for 45 s. 650 μl of SOC medium were added to each microfuge tube. The plasmid DNA/BL-21(DE3) mixture and the SOC medium were placed in a 37°C incubator, shaken at 225 r.p.m. for a 1 h and subsequently plated onto an agar substrate containing 100 μ g ml 87,221 ampicillin. The plates were then placed in a stationary incubator at 37°C for 12 h. Start-up cultures were prepared by inoculating individual colonies from the plasmid transformation plate into 25 mL of Luria-Bertani medium (LB) and 100 μg ml^−1^ of ampicillin. This culture was incubated at 37°C while shaking at 225 r.p.m. overnight. The start-up LB cultures were added to 1 L Terrific broth (TB) to maximize bacterial cell yield. TB cultures were incubated at 37°C, with shaking at 225 r.p.m. until OD600 reached 1.3, at which point 400 μl of isopropyl β-D-1-thiogalactopyranoside (IPTG) was introduced to induce protein expression. Prior to IPTG induction, a 500 μ l sample of un-induced cells was set aside for PAGE electrophoresis. TB cultures were incubated for a 12 h period at 20°C for a 3–4 h period at 37°C, with shaking at 225 r.p.m. and subsequently spun down at 7200 r.p.m. in a centrifuge at 4°C. A post-induction gel electrophoresis sample was taken prior to centrifugation to confirm heterologous expression. Cell pellets were collected, flash-frozen in liquid nitrogen, and stored at −80°C until lysis.

### Purification

Following SDS-PAGE verification of heterologous protein induction in bacteria, cell cultures were thawed at 0°C, suspended in a lysis buffer (30 mM Tris buffer, pH 8.0, containing 500 mM NaCl), vortexed, and then lysed at 20,000 psi in a Microfluidics M-100EH microfluidizer. Lysed cells were centrifuged at 42,000 r.p.m. for 45 min at 4°C; the supernatant was subsequently collected and bound to Ni-NTA resin for 12 h. Glutaredoxin-bound Ni-NTA resin was washed with 30 mM Tris buffer, pH 8.0, containing 500 mM NaCl plus 30 mM imidazole until the OD_280_of the eluent was ≤0.010. Glutaredoxin A was eluted from the column with 40–50 ml of 30 mM Tris buffer, pH 8.0, containing 300 mM imidazole, plus 500 mM NaCl. Post-elution protein purity was confirmed by SDS-PAGE using a Phastgel apparatus (GE Healthcare). Protein concentrations were determined either using the BioRad Protein Assay with bovine serum albumin as a standard (Bradford, 1976), or by using reference parameters calculated from the molecular weight and calculated extinction coefficient of the mutant in question (Gill and Von Hippel, 1989). Glutaredoxin A mutants were concentrated to approximately 6 mg/ml and further purified by gel filtration on a Superdex 75 column (5 × 300 mm). Protein was re-concentrated for crystallization using a 10 K cutoff Amicon spin concentrator.

### Crystallization

Crystallization trials of all mutant glutaredoxin proteins utilized the hanging-drop vapor-diffusion method. Noting previously identified glutaredoxin A wild-type crystallization conditions, 24 well trays were set up with a horizontally varying salt gradient (1.1–1.5 M ammonium sulfate), and a vertically varying pH gradient (pH 7.0–8.2 HEPES buffer). 1% PEG400 (w/v) was included throughout. Crystal droplets consisting of 2 μl protein solution and 2 μl reservoir solution were added and trays were placed in 23°C for crystallization. Long, rod-like structures displaying the typical morphology of glutaredoxin crystals generally appeared after 24 h; however, some mutants grew crystals in as much as 2 weeks.

### Structure solution

The crystals were captured on nylon loops and flash frozen in liquid N_2_. Initial data sets were collected on a Rigaku ScreenMachine. Subsequent data sets were collected at SLAC beamline 7-1. The data were collected at 90 K. X-ray data was processed with imosflm (Battye et al., 2011) and the data were scaled using SCALA as a part of the CCP4 package (Winn et al., 2011). 10% of the data were allocated for R-free cross validation. A summary of the crystal statistics are presented in Table 2.

Structures of all mutants were solved by molecular replacement using 3QMX as the target structure. We used the Phaser module as implemented in the Phenix package for this calculation. Subsequent electron density for the protein was manually fit using Coot (Emsley et al., 2010). Electron density assigned as water molecules was automatically assigned by Phenix during the final stages of refinement. Each water molecule was checked manually in Coot, and in cases that were missed by Phenix, waters were added manually to density that met the H-bonding geometry and inter-atomic distance criteria established by Phenix. Structural refinement was also performed using the Phenix package (Adams et al., 2010).

### Mutant analysis

The thermodynamic stability for each single site mutant in our data set was predicted using the SDM web server (Worth et al., 2011). This website calculates changes in stability that likely result from single site mutations in proteins. Factors such as the physiochemical differences between the wild-type and mutant protein within the known protein structure are used to compute a free energy term (Worth et al., 2011). A significant change in effective free energy correlates with either with a stabilizing or a destabilizing prediction (Table 1).

Potential conflicts with the wild-type crystal packing were analyzed in Pymol (Schrodinger, 2013). First, the wild-type residue of interest was selected in Pymol. Then, complete symmetry-related molecules in crystal lattice were expanded in a sphere 5 Å around that amino acid. All of the residues within 5 Å of the amino acid were selected and counted. Simulated mutants were computed using the Pymol mutagenesis wizard. The most common rotamer was selected without any subsequent energy minimization. The same symmetry expansion and residue selection was performed as was described from the wild-type protein. The net difference between the number of neighboring residues in the wild-type protein and the mutant protein was reported as Δ # Neighbors (Table 1).

### Conflict of interest statement

The authors declare that the research was conducted in the absence of any commercial or financial relationships that could be construed as a potential conflict of interest.
